# Osteogenically-induced exosomes stimulate osteogenesis of human adipose-derived stem cells

**DOI:** 10.1007/s10561-020-09867-8

**Published:** 2020-11-20

**Authors:** Mengru Zhu, Yang Liu, Hongzhi Qin, Shuang Tong, Qiang Sun, Ting Wang, Hua Zhang, Mengying Cui, Shu Guo

**Affiliations:** 1 Department of plastic surgery, The First Affiliated Hospital of Dalian Medical University, 222 Zhongshan Road, Dalian, 116011 China; 2 School of Chemical Engineering, Dalian University of Technology, No. 2 Linggong Road, Dalian, 116024 China; 3 Department of Plastic surgery, The First affiliated Hospital of China Medical University, No 155 Nanjing North Street, Shenyang, 110002 China

**Keywords:** Human adipose-derived stem cells, Exosomes, Osteogenic differentiation, Bone tissue engineering

## Abstract

Exosomes exhibit great therapeutic potential in bone tissue engineering. The study aimed to investigate whether the exosomes derived from human adipose-derived stem cells (hADSCs-Exos) during different time-span of osteogenic differentiation could promote osteogenesis. The appropriate concentrations of hADSCs-Exos to enhance the proliferation, migration and osteogenesis of hADSCs-Exos were also examined. PKH67 labelled hADSCs-Exos was used to detect the internalization ability of hADSCs. The osteogenic differentiation abilities of hADSCs after treatment with hADSCs-Exos was evaluated by Alizarin red staining (ARS). The proliferation and migration of hADSCs was examined by cell counting kit-8 and wound healing assay, respectively. The expression of exosomal surface markers and osteoblast-related protein of hADSCs was assessed by Western blot. PKH67-labelled exosomes were internalized by hADSCs after 4 h incubation. ARS showed that the amount of mineralized nodules in Exo^1−14d^ group was significantly higher than that in Exo^15−28d^ group. hADSCs-Exos could promote the proliferation and migration capacity of hADSCs. Western blot analysis showed that after hADSCs-Exos treatment, ALP and RUNX2 were significantly enhanced. Specially, the Exo^1−14d^ group of 15 μg/mL significantly upregulated the expression of RUNX2 than the other exosomes treated groups. Our findings suggest that exosomes secreted by hADSCs during osteogenic induction for 1–14 days could be efficiently internalized by hADSCs and could induce osteogenic differentiation of hADSCs. Moreover, administration of Exo^1−14d^ at 15 μg/mL promoted the proliferation and migration of hADSCs. In conclusion, our research confirmed that comprised of hADSCs-Exos and hADSCs may provide a new therapeutic paradigm for bone tissue engineering.

## Introduction

Globally, a large number of people have suffered from bone defects owing to trauma, infection osteonecrosis, congenital deformities, resection of tumors and other bone diseases (Chen et al. 2010; Seong et al. 2010). To date, bone grafting is considered as the “gold standard” for treatment of bone defects, including autologous bone grafts, allogeneic bone grafts, and bone-graft substitutes (Dimitriou et al. 2011). However, all these techniques have certain limitations, such as donor-site defect, poor bone quality and limited availability of grafting material (Du et al. 2019; Lord et al. 1988).

Recently, the remarkable development of bone tissue engineering has brought the dawn to the therapy of bone defects (El-Rashidy et al. 2017). Mesenchymal stem cells (MSCs) are a population of self-renewing multipotent cells that have been proposed as promising candidates for regenerative medicine (An et al. 2019; Xie et al. 2016; Zuk et al. 2002), especially, bone marrow-derived mesenchymal stem cells (BMSCs), are regarded as one of the most widely applied stem cell with great clinical potential for regenerative medicine and cell-based therapies. Osteogenic differentiation of BMSCs facilitate the recovery of osteoporosis has been widely reported (Huo et al. 2018; Oryan et al. 2017). Nevertheless, BMSCs exert drawbacks that may hinder their clinical applications with regards to insufficient cell number and complex harvesting procedures. Alternatively, human adipose-derived stem cells (hADSCs) are considered an ideal cell source (Kim et al. 2007; Schaffler and Buchler 2007), which can be extensively extracted from discarded adipose tissue. Compared with BMSCs, ADSCs have the following advantages, including abundant sources, easy access, low immunogenicity, rapid proliferation with multilineage potential and few ethical concerns (An et al. 2019). More importantly, the osteogenic capacity of hADSCs appears to be minimally affected by aging, which enhancing the clinical applicability for the gerontal patient (Liao and Chen 2014; Shi et al. 2005; Wu et al. 2013).

Emerging evidences have shown that hADSCs can differentiate into osteoblasts participating the bone regeneration in vivo and in vitro (Chen et al. 2010; Xu et al. 2017). They promote osteogenesis mainly by two approaches. Firstly, differentiated hADSCs to target cells that participate in osteogenesis; secondly, differentiated hADSCs secrete a numerous of cytokines and extracellular vesicles (EVs) taking part in cell-to-cell communication via paracrine manner to promote cell proliferation, migration of various cell lines that are mandatory to promote osteogenesis. The latter has been confirmed to contribute more significantly than the capacity of directly differentiating in tissue regeneration (Ma et al. 2019; Phinney and Prockop 2007).

EVs released from hADSCs are involved in tissue regeneration and contribute to the paracrine effect of hADSCs (Cooper et al. 2018; Liu et al. 2020; Wong et al. 2019). Exosomes are a type of EVs with a diameters range between 30 and 150 nm (Mathivanan et al. 2010; Tkach and Thery 2016), containing pivotal functional biomolecules (DNA, proteins, mRNA, microRNA and lipids) which modulate biological behavior by horizontally transferring to recipient cells (Hoshino et al. 2015). Current research about exosome exhibit a broad range of therapeutic potentials, including cancer, regenerative medicine and immune disorders (Shimasaki et al. 2018; Urbanelli et al. 2015), and it is clear that exosomes are an important avenue in the field of bone regeneration (Fang et al. 2019; Li et al. 2018b; Qin et al. 2016). The application of hADSCs-derived exosomes (hADSCs-Exos) may provide a novel strategy for bone tissue engineering and regeneration, since it has been found that hADSCs-Exos could promote BMSCs osteoinductive capacity under the condition of supplementing osteogenic medium in vivo and in vitro (Li et al. 2018a). Nevertheless, considering prominent potential of hADSCs in osteogenesis, the further research of effects on combination of hADSCs-Exos with hADSCs for bone regeneration is required. Therefore, it is of great significance and value to evaluate the appropriate stage and concentration of exosomes during the osteoinductive process.

In the present study, we aimed to determine whether exosomes from hADSCs during osteogenic induction become internalized by target hADSCs and influence hADSCs proliferation, migration and osteogenic differentiation in a stage-and concentration-dependent manner. The combination application of hADSCs and hADSCs-Exo is expected to provide new ideas for stem cell therapy to stimulate osteogenesis.

## Materials and methods

### Isolation and culture of hADSCs

This study was approved by the Research Ethical Committee of the First Hospital of China Medical University. Human abdominal subcutaneous adipose tissue acquired with informed consent were digested by 0.1% type I collagenase (Worsington, USA) in a 37 °C water bath for 45 min. The digestion was terminated using hADSCs culture medium (Cyagen Biosciences, USA) containing 10% fetal bovine serum (FBS, Gibco, USA) and was centrifuged at 1200 rpm for 5 min. The cell pellet was then suspended in hADSCs proliferation medium (PM, hADSCs culture medium containing 10% FBS, 1% penicillin-treptomycin, and 1% glutamine [Cyagen Biosciences, USA]) and cultured into a 75-cm^2^ cell culture flask (Corning, USA). The cells were incubated at 37 °C in 5% CO_2_ incubator (Thermo Forma, USA), and were passaged until passage 4 for subsequent experiments.

### Characterization of hADSCs

The multi-differentiation of hADSCs was induced by adipogenic, osteogenic and chondrogenic induction medium (all from Cyagen Biosciences, USA), respectively. The adipogenic induction was confirmed by Oil red O staining at day 14, the calcium mineral deposits were measured by Alizarin red staining (ARS) at day 21, and the chondrogenic induction was confirmed by Alcian blue staining at day 28 (all from Cyagen Biosciences, USA). An optical microscope (Olympus, Tokyo, Japan) was used for observation and image capture.

The expression of cell surface antigens was analyzed by flow cytometry. Cell suspension was collected and centrifuged at 1000 rpm for 5 min, washed with phosphate-buffered saline (PBS, Gibco, USA). The CD34, CD90 and CD105 antibodies (BD Bioscienses, USA) were added to the cells and incubated at room temperature for 30 min in the dark. The cell suspension was then washed with PBS and detected by flow cytometer (BD, USA, Aria II). The cells without labelling were regarded as control group.

### hADSCs-Exos extraction and identification

Exosomes were isolated from conditioned medium of hADSCs in vitro. The exosomes-depleted FBS, obtained by ultracentrifugation at 100,000 g for 3 h as previously described (Nabet et al. 2017), was used in all conditions in this study. To induce osteogenic differentiation, hADSCs were seeded in osteogenic induction media (OM) for 28 days. Exosomes from different time-span were isolated from supernatants of undifferentiated hADSCs in PM (Exo^0d^), and the differentiated hADSCs during day 1 to 14 (Exo^1−14d^) and day 15 to 28 (Exo^15−28d^), and purified by sequential centrifugation. The conditioned medium was collected every 3 days and centrifuged at 2000 g for 30 min, and then at 20,000 g in a sterile Ultra-Clear™ tube (Beckman Coulter, USA) for 1 h before being passed through a 0.22 μm filter (Millipore, USA) to eliminate some large particles. Afterward, the filtered solution was then ultracentrifuged at 100,000 g for 1 h. Then, the exosomes were washed with PBS and again pelleted at 100,000 g for 70 min to remove protein contamination. Finally, the pelleted exosomes were resuspended in PBS. All procedures were performed at 4 °C.

For exosomes identification, transmission electron microscopy (TEM) was performed to monitor the morphology of exosomes. In brief, 10 μL of Exosomes (Exo^0d^, Exo^1−14d^ and Exo^15−28d^), were fixed with 4% neutral paraformaldehyde (Sigma, USA) at room temperature for 20 min by an Ultrasonic cleaning apparatus (KQ-250, KunShan, China). Thereafter, the fixed exosomes were dropped onto carbon-coated electron microscope copper mesh, and air-dried for 15 min. Micrographs were obtained using a JEM-2000EX TEM (JEOF, Japan). Dynamic light scattering (DLS) was used to evaluate the size of hADSCs-Exos. Briefly, 150 μL of exosomes suspension was diluted in PBS and dispersed at 70 kw for 30 min at room temperature. The hydrated particle size was determined by laser doppler micro-electrophoresis (Malvern Nano ZS, UK). Finally, the markers (CD9 and CD63) expression was detected by Western blot (all from abcam, UK). Exosome protein was quantified using the bicinchoninic acid assay kit (Solarbiol, China). Total protein was examined to confirm the purity of the exosomes.

### Exosomes labelling and confocal microscopy

For exosomes internalization assay, Exo^0d^, Exo^1−14d^ and Exo^15−28d^ were labelled with PKH67 Green Fluorescent Cell Linker Kit (Sigma, USA). Firstly, 15 μg exosomes suspended in 500 μL Dilut C, mixed with equal volume of PKH67 dye were incubated at 37 °C for 8 min. The labelling reaction was terminated by ultracentrifugation at 100,000 g for 1 h and resuspended in PBS. Thereafter, the labelled exosomes were incubated with serum-free hADSCs with seeding intensity of 2 × 10^4^ cells/cm^2^ in 24-well plate in dark for 4 h. The preparations were then fixed with 4% paraformaldehyde for 30 min, after that, the cell nucleus were stained with 6-Diamidino-2-phenylindole (DAPI, Sigma, USA) for 20 min. Exosomes internalized by hADSCs was visualized with confocal microscope (Leica SP8, Germany).

### Exosomes treatment of hADSCs and ARS

To determine the optimal time-span of extracted hADSC-Exos during hADSCs osteogenic differentiation, the hADSCs of passage 4 were seeded into the 24-well plates, and was cultured in PM. After getting 70–80% confluence, the culture medium was replaced by PM containing exosomes (Exo^0d^, Exo^1−14d^ and Exo^15−28d^) from different time-span of hADSCs osteogenesis induction. hADSCs cultured in exosomes-free PM and OM were set as negative and positive controls, respectively.

The mineralized deposits were evaluated by ARS after incubation for 21 days. After fixation by 4% paraformaldehyde for 30 min, the cells were washed three times with PBS before staining with Alizarin red S for 5 min, and then the samples were washed again with PBS. For ARS quantitation the mineralized deposits were dissolved in 10% cetylpyridinium chloride (Sigma, USA) in dark for 30 min at room temperature. The solution was transferred to a 96-well plate with 100 μL/well. The optical density (OD) values was measured at 562 nm using a microplate reader (BioTek EL808, USA).

### Cell proliferation assay

On the basis of the ARS results, Exo^1−14d^ was selected as the optimal time-span for hADSCs-Exos isolation during hADSCs osteogenic induction in the following experiments. Cell Counting Kit-8 (CCK-8) assay (Dojindo, Kyushu Island, Japan) was carried out to the detect the optimal concentration of hADSC-Exos on the proliferation of hADSCs. In brief, hADSCs were seeded into 96-well plate (5 × 10^3^ cells/well) in PM without serum. Subsequently, when the cells grown into 60% confluence, the cells were exposed to PM containing different concentrations of Exo^1−14d^ (10, 15 and 20 μg/mL) at 100 μL/well for 24, 48 and 72 h. Next, CCK-8 solution (10 μL/well) was added to each well and incubated in dark at 37 °C for 3 h. The absorbance at 450 nm was measured by spectrophotometric methods. Each experiment was repeated for at least 3 times.

### Cell migration assay

Cell migration was investigated with wound healing assay. Firstly, hADSCs were seeded into 24-well plates (2 × 10^4^ cells/cm^2^) and incubated with PM. After the cells fully attached, the confluent monolayer was scratched by a sterile pipette tip. Each well was washed with PBS to remove the cellular debris, and then supplemented with 1 mL of non-serum conditioned medium containing 10, 15 and 20 μg/mL of Exo^1−14d^. The cells were photographed at 0, 12, and 24 h using microscope (Leica DMI4000B, Germany). The migration area ratio was measured by Image J software (National Institutes of Health, USA). The migration area ratio = (A_0_ − A_n_)/A_0_, where A_0_ represents the initial wound area (t = 0 h), and A_n_ represents the residual area of the wound at the assess point (t = n h).

### Alkaline phosphatase (ALP) staining and activity analysis

After hADSCs were incubated with 10, 15 and 20 μg/mL of Exo^1−14d^ for 7 and 14 days, the ALP staining was performed using a BCIP/NBT ALP staining Kit (Beyotime Technology, China) according to the manufacturer's instructions. The cells were incubated at room temperature for 2 h in dark, and washed twice with distilled water. The samples were observed by microscope (Leica DMI4000B, Germany). Meanwhile, ALP activity was evaluated using an ALP Assay Kit (Beyotime Technology, China). In brief, the cells were lysed by 1% Triton X-100 (Sigma, USA), and then centrifuged at 1200 rpm for 10 min. The ALP activity was measured by detecting the OD at 405 nm.

### Western blot analysis

Western blot was conducted to measure the exosomal and osteoblast-related proteins. hADSCs were seeded with PM in 6-well plate (2 × 10^4^ cells/cm^2^). Until reaching 80% confluence, the cells were incubated with Exo^1−14d^ at concentrations of 10, 15 and 20 μg/mL for 14 days. The cells or exosomes were lysed with RIPA lysis buffer along with the Protease inhibitor (Sigma-Aldrich, USA). Protein extracts were directly heated at 100 °C for 7 min in  × 5 loading buffer (Life Technology, USA) and separated on 10% sodium dodecyl sulfate–polyacrylamide gel electrophoresis (SDS-PAGE) gels (Solarbiol, China) then transferred to the polyvinylidene difluoride (PVDF) membrane (Solarbiol, China). The membranes were blocked with 5% non-fat milk, and incubated at 4 °C overnight with primary antibodies CD9 and CD63 (1:500, Abcam, UK), anti-RUNX 2, and anti-ALP (1:200, Santa Cruz), respectively. Then the membranes were incubated with goat anti-mouse secondary antibodies (1:1000, Abcam, UK). The results were visualized using enhanced chemiluminescence reagent (Beyotime Biotechnology, China) and imaged by Image Analysis System (Tanon, Japan).

## Statistics analysis

All data are expressed as mean ± SD, and performed in triplicate. Differences between groups were analyzed by one-way analysis of variance (ANOVA). To compare the samples pair-to-pair, the Bonferroni Test or LSD Test was used when the variance was homogeneous otherwise the Dunnett’s T3 Test was used in this study. SPSS 20.0 was used for all statistical analysis and differences were considered to be statistically significant as a result of *p* < 0.05.

## Results

### Characterization of hADSCs

**Fig. 1:**
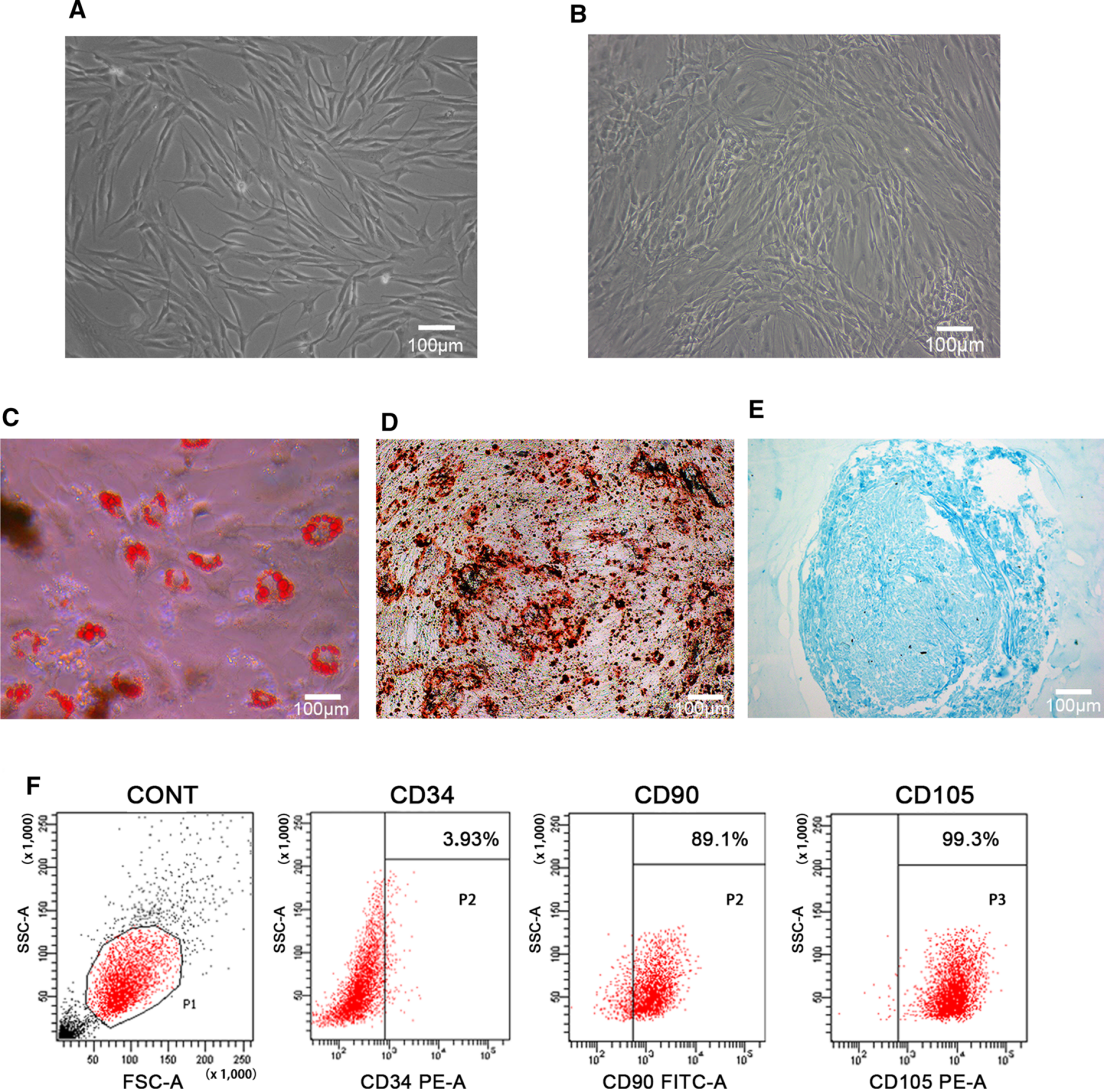
Isolation and characterization of human adipose-derived stem cells (hADSCs). **A** Primary hADSCs presented a long spindle shape; **B** hADSCs of passage 4 showed vortex pattern; **C** Oil red staining of hADSCs after adipogenic induction for 14 days; **D** Alizarin red S staining of hADSCs after osteogenic induction for 21 days; **E** Alcian blue staining of hADSCs after chondrogenic induction for 28 days; **F** Characterization of hADSCs by flow cytometry. The cells without labelling were regarded as control group. Positive antigens: CD90, CD105; negative antigen: CD34. (Color figure online)

Morphology of hADSCs was observed by inverted microscope. Primary hADSCs substantially adhered to the dish for 48 h, and then displayed typical fibroblastic-shaped or polygon in morphology (Fig. 1A). It can be seen that with the increase of the cell density the hADSCs exhibited a vortex distribution at passage 4 (Fig. 1B).

To evaluate multilineage differentiation of hADSCs, the cells of passage 4 were incubated in adipogenic, osteogenic and chondrogenic induction medium, respectively. The oil red staining results after incubation in adipogenic medium for 14 days showed that there were aggregated lipid droplets (Fig. 1C). After incubation for 21 days, ARS results indicated that there were a large number of calcified nodules (Fig. 1D). Alcian blue staining results showed a blue internal acid mucopolysaccharide in the cartilage tissue after incubation in chondrogenic induction medium for 28 days (Fig. 1E).

Flow cytometry analysis of hADSCs of passage 4 confirmed that cells were positive for CD 90 (89.1%), CD105 (99.3%), and were negative for CD34 (3.93%) (Fig. 1F).

### Identification of hADSCs-Exos

**Fig. 2:**
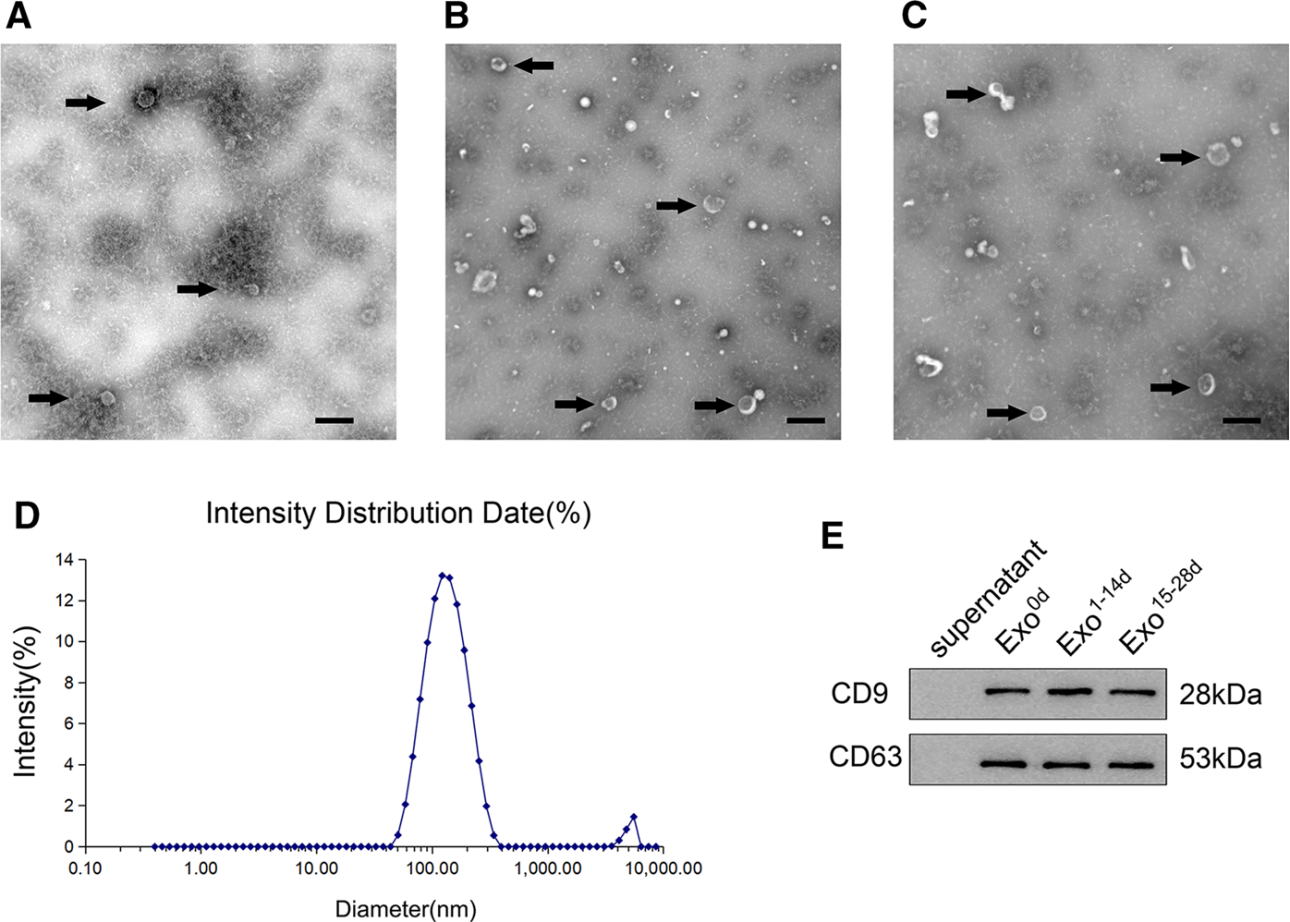
Extraction and identification of exosomes derived from human adipose-derived stem cells (hADSCs-Exos) during different time-spans of osteogenesis induction. The representative images of hADSCs-Exos during time-span of osteogenesis induction at Day 0 (Exo^0d^, **A**), Day 1 to 14 (Exo^1−14d^, **B**), and Day 15 to 28 (Exo^15−28d^, **C**) were observed by transmission electron microscopy (TEM). Black arrows indicated the exosomes. Scale bar = 200 nm. **D** The particle size distribution of hADSCs-Exos was measured by dynamic light scattering (DLS) analysis, and the mean size of hADSCs-Exos was 125.8 ± 3.822 nm. **E** Western blot analysis showed that the specific surface markers of exosomes (CD9 and CD63) were detected

TEM showed that exosomes extracted from conditioned medium of hADSCs proliferation and different time-span (Exo^0d^, Exo^1−14d^ and Exo^15−28d^) of osteogenic induction possessed typical cup-shaped morphology. The morphology of exosomes in Exo^0d^ group (Fig. 2A) was uniform and round. While exosomes with different sizes in Exo^1−14d^ and Exo^15−28d^ group are detectable (Fig. 2B, C). DLS results showed different groups of exosomes of size range, with the average diameter of 125.8 ± 3.822 nm, and the particle size distribution coefficient (PDI) is 0.282 (Fig. 2D). Western blot analysis showed that hADSCs-Exos expressed exosomal surface markers CD9 and CD63 (Fig. 2E). As our previous studied (Yang et al. 2019; Zhu et al. 2019), all these indicated the extracted exosomes was in accordance with the standard.

### Internalization of exosomes in hADSCs

**Fig. 3:**
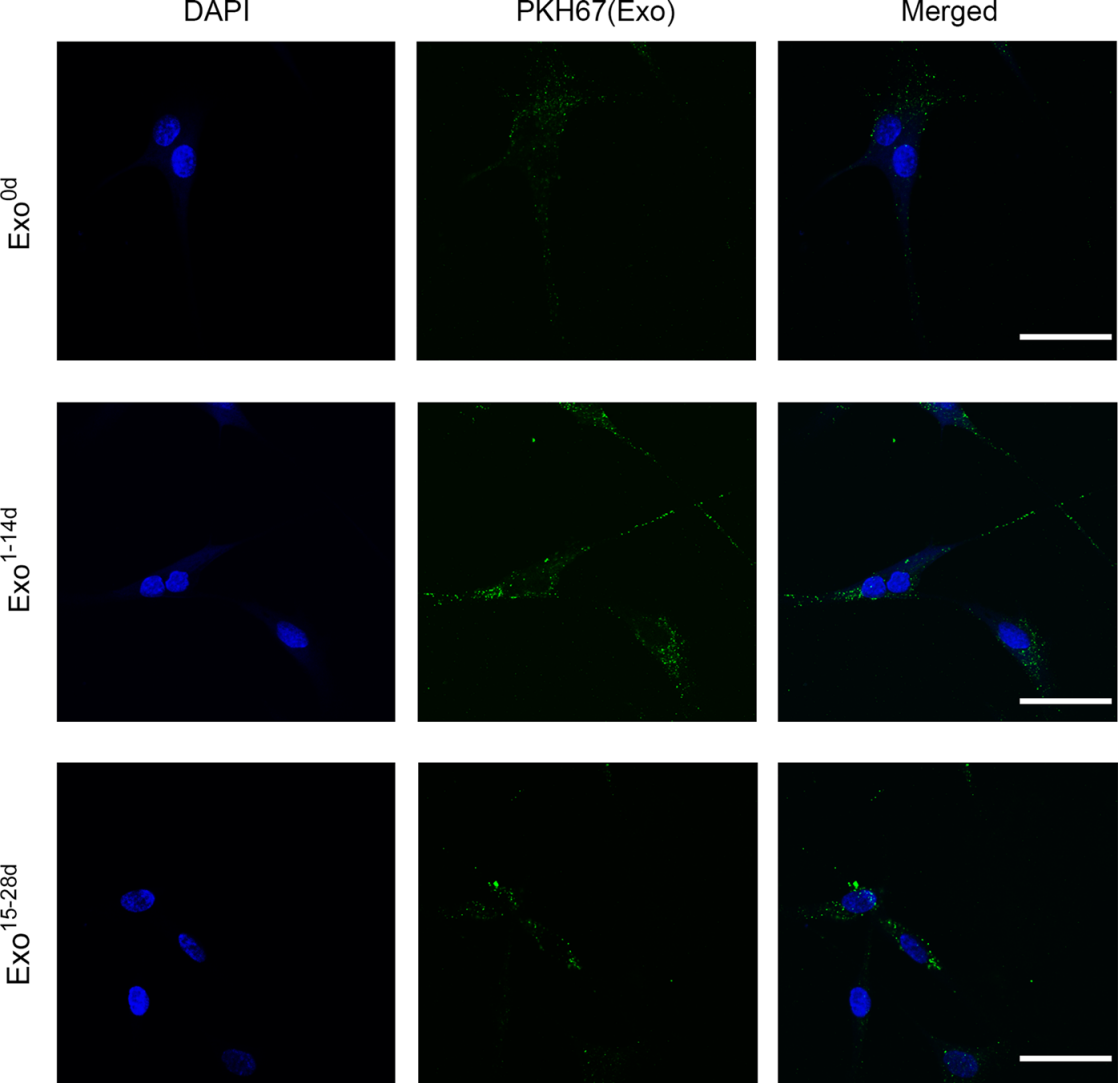
Internalization of exosomes in hADSCs. The PKH67-labelled exosomes (Exo^0d^, Exo^1−14d^, and Exo^15−28d^, green) from hADSCs were detected in the cytoplasm of hADSCs after incubation for 4 h by confocal microscopy, respectively. The nucleus of hADSCs were stained with DAPI (blue). Scale bar = 50 μm. (Color figure online)

To investigate whether hADSCs-Exos could enter into the hADSCs, the PKH67-labeled hADSCs-Exos (Exo^0d^, Exo^1−14d^ and Exo^15−28d^) were co-cultured with native hADSCs for 4 h, and evaluated by confocal microscopy analysis. The images demonstrated that the exosomes (green) were internalized by hADSCs and distributed in the cytoplasm. Moreover, it is revealed that hADSCs-Exos tended to accumulate in the perinuclear region (Fig. 3).

### Optimal time-span of hADSCs-Exos for hADSCs osteogenic differentiation

**Fig. 4:**
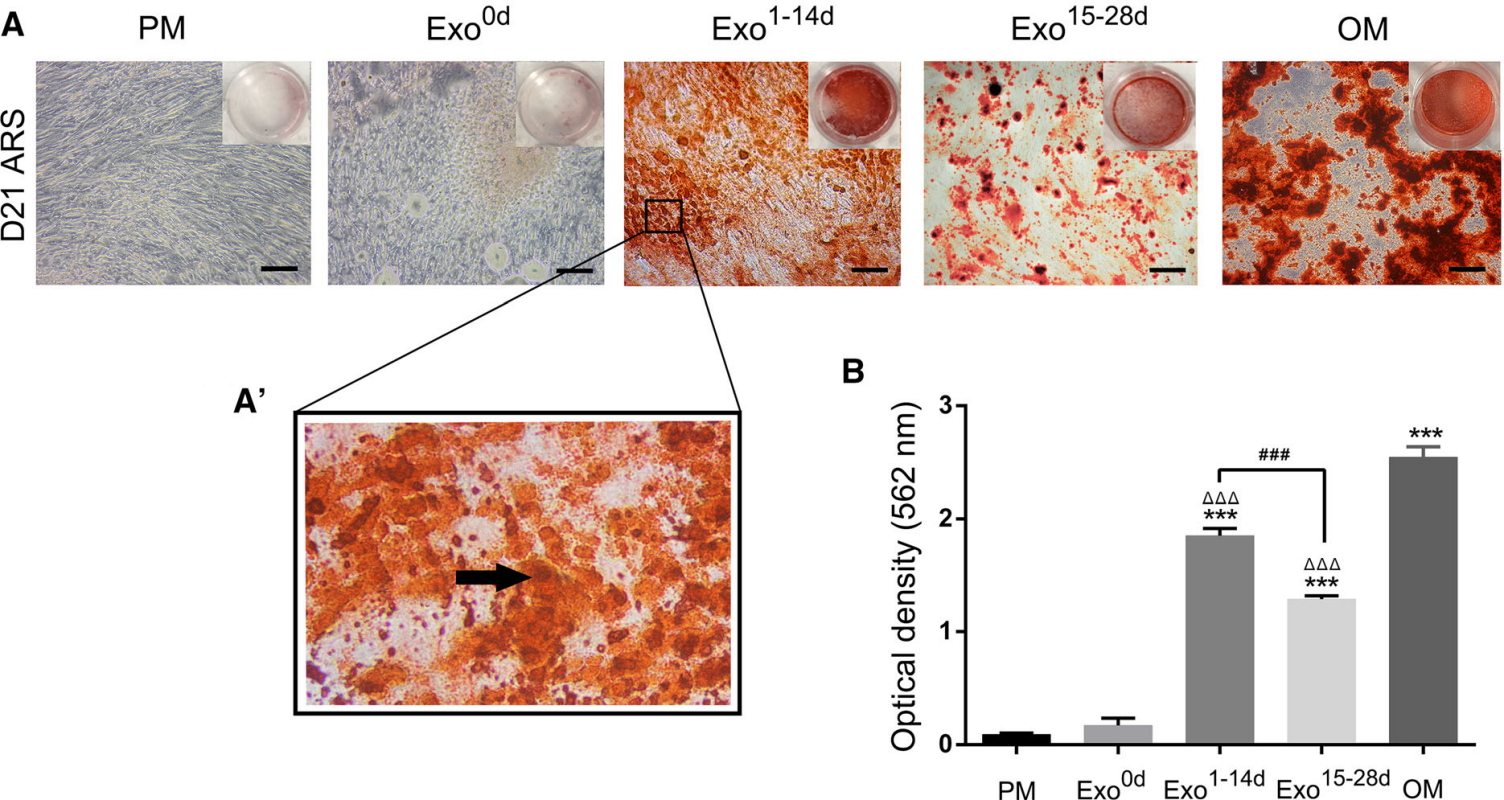
Alizarin red S staining (ARS) assays of hADSCs after osteoinduction to determine the optimal time-span for exosomes. **A**: ARS staining of hADSCs incubated with PM, OM and different time-span of osteogenic induction exosomes (Exo^0d^, Exo^1−14d^ and Exo^15−28d^) on day 21. Scale bar = 200 μm. **A’** The higher magnification panels of black box in Fig. 4A. The black arrow indicated the calcified nodules. **B** Quantification of ARS of hADSCs treated with or without exosomes. *PM*: proliferation medium; *OM*: osteogenic medium. ***p < 0.001 compared with PM group, ^ΔΔΔ^*p* < 0.001 compared with OM group, ^###^*p* < 0.001 represents significant differences between compared groups. (Color figure online)

To study the osteoinductive activity of the exosomes isolated from hADSCs at different time-span of osteogenic induction, ARS staining was used as an indicator. The ARS (Fig. 4A) and quantitative analysis of calcium nodule (Fig. 4B) showed that after co-culturing for 21 days, only very few mineralized nodules were formed in the negative group (PM) and Exo^0d^ group (A_PM_ = 0.091 ± 0.012, A_Exo0d_ = 0.172 ± 0.064), while the higher magnification panels of calcified nodules (A_Exo1-14d_ = 1.851 ± 0.064) were observed in Exo^1−14d^ group (Fig. 4A). Moreover, only a small amount of dispersed mineralized nodules (A_Exo15-28d_ = 1.290 ± 0.003) were observed in Exo^15−28d^ group. The calcium nodule content in the OM group (A_OM_ = 2.544 ± 0.091) was the highest among all the groups. There were significantly more calcium nodules in the Exo^1−14d^ group than that in PM and Exo^15−28d^ (*p* < 0.001). The above results indicated that Exo^1−14d^ could promote osteogenic differentiation. Consequently, time-span of day 1–14 during osteogenic induction (Exo^1−14d^) was used in the following studies.

### Effects of hADSCs-Exos on hADSCs proliferation, migration and osteogenic differentiation

**Fig. 5:**
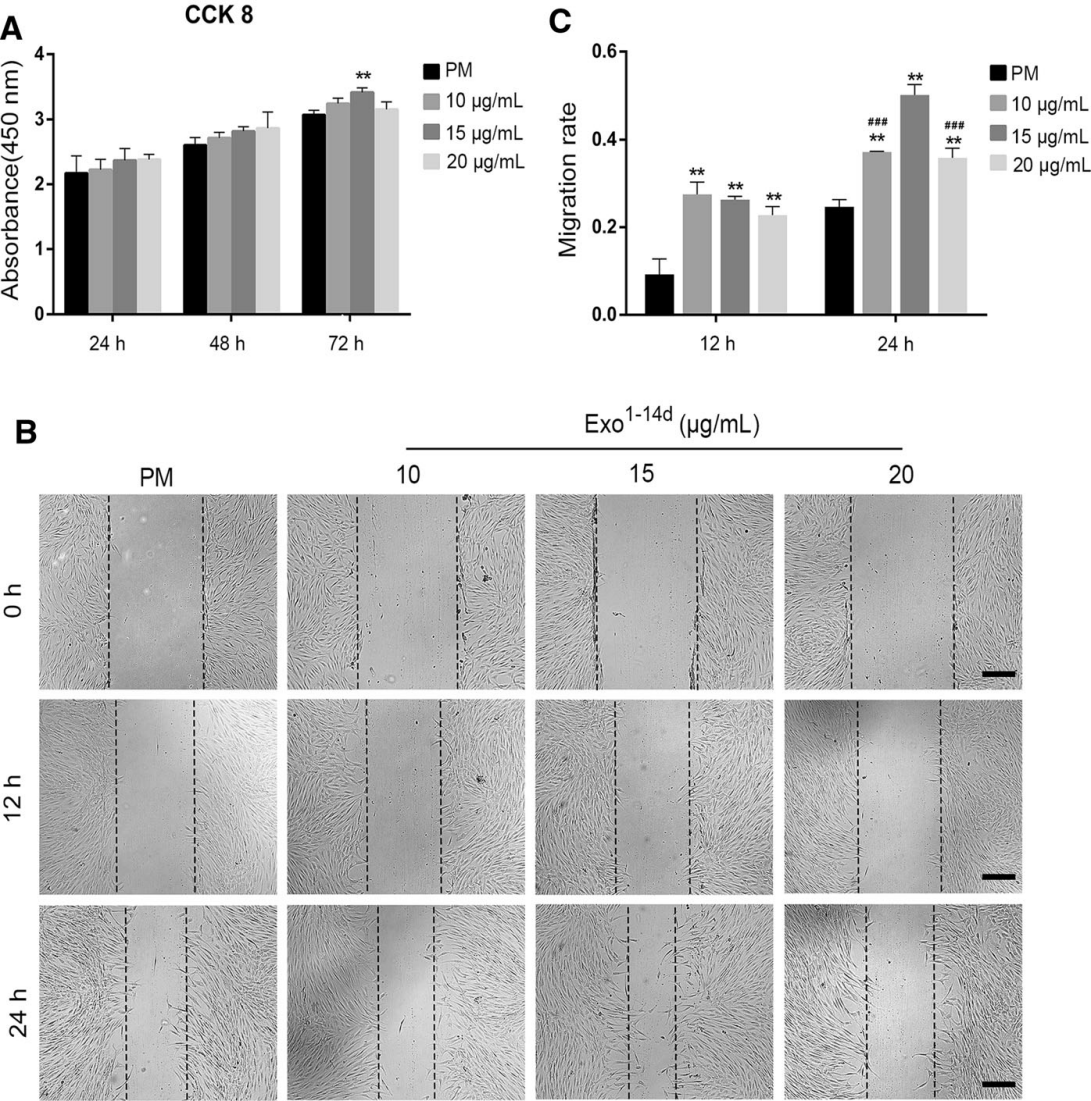
hADSCs-Exos enhanced the proliferation and migration of hADSCs. **A** CCK-8 analysis of hADSCs after treatment with exosomes (10, 15 and 20 μg/mL) from hADSCs after 14 days of osteogenic differentiation (Exo^1−14d^) for 24, 48, and 72 h. **B**, **C** Migration of hADSCs co-cultured with different concentration of Exo^1−14d^ (10, 15 and 20 μg/mL) assessed by wound healing assay in 0 h, 12 h and 24 h. Scale bar = 200 μm. The quantitative results of wound healing assay.*PM*: proliferation medium; **p < 0.01 compared with PM group, ^###^*p* < 0.001 compared with 15 μg/mL Exo^1−14d^ group

To explore the effects of hADSCs-Exos on hADSCs proliferation, cells were treated with Exo^1−14d^ at concentrations of 10, 15 and 20 μg/mL for 24, 48 and 72 h using CCK-8 method. As shown in Fig. 5A, cell viability was enhanced in a concentration-and time-dependent trend after treatment for 24 and 48 h. However, there were no significant differences among all groups. After treatment with hADSCs-Exos for 72 h, only cells treated with Exo^1−14d^ at a concentration of 15 μg/mL showed significantly greater proliferation than that without hADSCs-Exos treatment (*p* < 0.01).

To examine whether hADSCs-Exos exhibited biological effects relevant to migration of recipient hADSCs, wound healing assay was performed. The results showed that hADSCs-Exos significantly promoted the migration of hADSCs (Fig. 5B, C, *p* < 0.01). However, the cell migration ability showed a decreasing trend with the increase of exosome concentration for 12 h, without significant differences (*p* > 0.05). Furthermore, after treatment with 15 μg/mL Exo^1−14d^ of for 24 h, cells exhibited greater migration ability than treated with 10 and 20 μg/mL (*p* < 0.001).

**Fig. 6:**
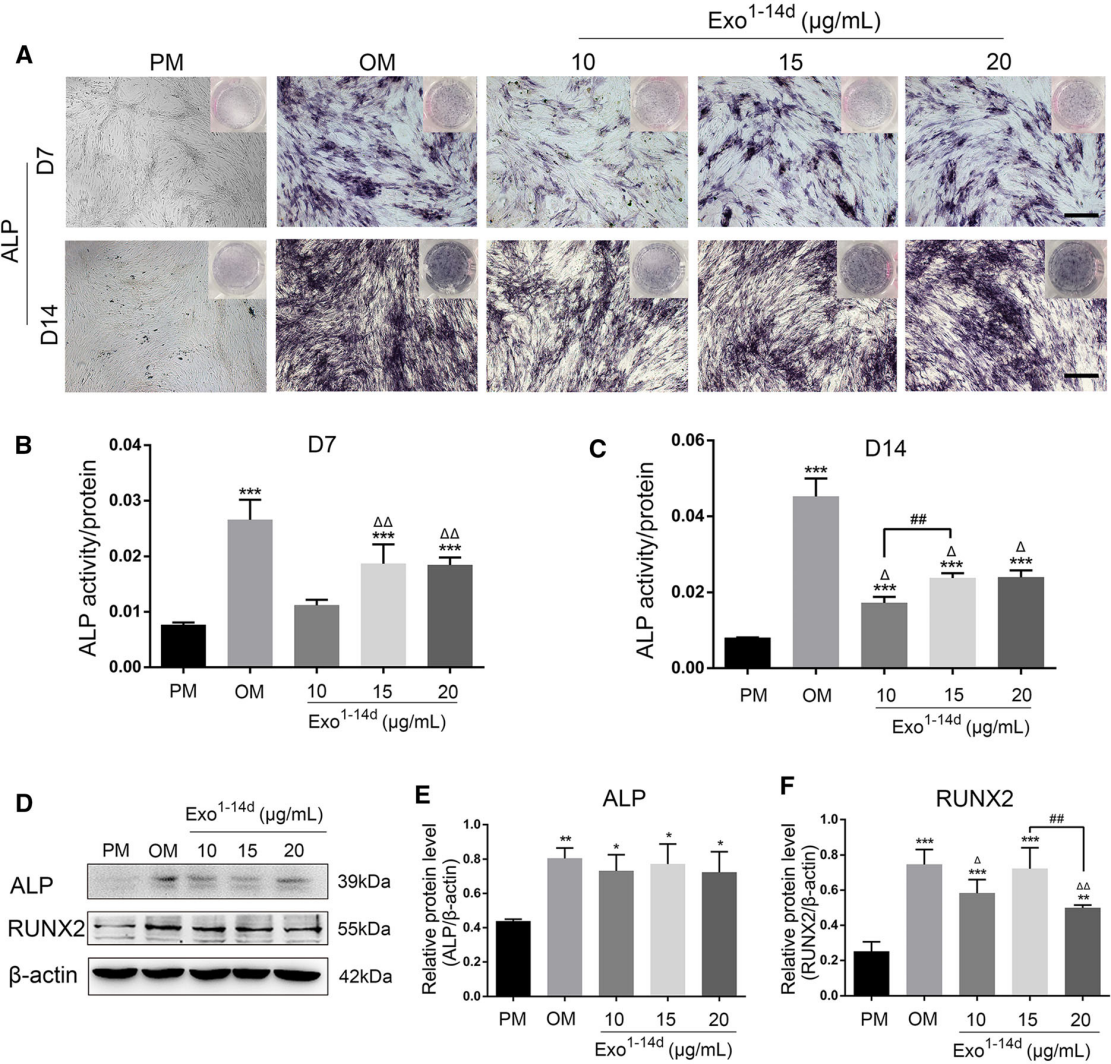
hADSCs-Exos promoted osteogenic differentiation of hADSCs in vitro. **A** Alkaline phosphatase (ALP) staining of hADSCs after incubation with exosomes (10, 15 and 20 μg/mL) from hADSCs after 14 days of osteogenic (Exo^1−14d^) on day 7 and day 14. Scale bar = 200 μm; **B**, **C** ALP activity normalized against the total protein level on day 7 and 14, respectively; **D** Western blot showed the expression of osteogenic-related proteins ALP and RUNX2 on day 14; **E**, **F** Relative protein level of ALP and RUNX2 on day 14, respectively. *PM*: proliferation medium; *OM*: osteogenic medium; *p < 0.05, **p < 0.01, and ***p < 0.001 compared with PM group, respectively. ^Δ^*p* < 0.05 and ^ΔΔ^*p* < 0.01 compared with OM group, respectively; ^##^*p* < 0.01 represents significant differences between compared groups

After 7 and 14 days of treatment with 10, 15 and 20 μg/mL of Exo^1−14d^, the ALP activity was determined as an indicator of osteogenesis (Fig. 6A–C). All experimental groups of the treated hADSCs revealed an elevation in staining and activity of ALP in comparison to that of PM without hADSCs-Exos. However, only treatments with 15 and 20 μg/mL Exo^1−14d^ (*p* < 0.001) induced a significant increase in ALP activity than that in the control group in PM at day 7. Moreover, all treatments with hADSCs-Exos had markedly greater ALP activity levels compared with PM at 14 days treatment (*p* < 0.001). The ALP activities in cells induced with the standard osteogenic medium (OM) was the highest among all groups.

Consistently, the expression of osteoblast-related proteins (ALP and RUNX2) in hADSCs was also significantly upregulated when treated with exosomes on day 14 in comparison to that of PM (Fig. 6D–F) (*p* < 0.05). No significant differences could be detected between the exosomes-treated group and the control group in OM in terms of ALP protein expression level. The expression of RUNX2 after treated with 15 μg/mL Exo^1−14d^ was significantly higher than that by 20 μg/mL Exo^1−14d^ (*p* < 0.01), and even no differences were found in comparison with the OM group.

## Discussion

MSCs have been widely studies as a promising candidate for regenerative medicine owing to their self-renewal capability and multilineage differentiation potential of osteogenesis, chondrogenesis, adipogenesis and neurogenesis (Huo et al. 2018; Majidinia et al. 2018). To date, hADSCs have become an attractive source in bone tissue engineering due to their stronger osteogenic differentiation ability compared with MSCs derived from the other sources, as well as easy acquisition and abundance in number (Damien and Parsons 1991; Kim et al. 2019; Rodriguez et al. 2005). In our study, hADSCs were extracted from adipose tissue obtained from healthy young female with abdominal liposuction. The primary hADSCs were isolated and characterized by surface antigen and multi-differentiation potentials.

Growing evidence showed that paracrine effect has been considered as the predominant mechanism for the role of MSCs in tissue repair (Li et al. 2018b). hADSCs, as an principal member of MSCs, participate in osteogenesis by this paracrine manner as well. Recent studies have revealed that during bone regeneration, hADSCs not only participate in osteogenesis via directly differentiation, but also by releasing a numerous of cytokines and EVs affecting this process (Ma et al. 2019). Recently, exosomes, as the typical EVs, appears to be a promising therapeutic strategy for bone regeneration (Li et al. 2018a; Phinney and Pittenger 2017). In comparison to direct autologous cell transplantation, exosomes-based therapy has advantages, such as precise targeting effect, high stability, easy to cross biological barriers, low immunogenicity, low toxicity and less immune responses (El Andaloussi et al. 2013; Li et al. 2018a). The application of hADSCs-Exos in tissue engineering and regenerative medicine has attracted great interest since a number of studies have demonstrated the therapeutic effects by hADSCs-Exos on wound healing and tissue repair in various physiological systems (Wang et al. 2018). A recent study revealed that exosomes from conditioned media of BMSCs promote bone regeneration during early stage, as well as enhanced angiogenesis, which contributes to the progression of osteogenesis (Takeuchi et al. 2019). Thus, our goal is to develop an effective exosomes-based therapeutic method to assist bone regeneration.

In the natural osteogenesis and bone regeneration, four distinct but sequential phases occur: lineage commitment, proliferation, matrix synthesis and mineralization (Javed et al. 2010; Matta et al. 2019). Since this process is a relatively independent and overlapping (Cano et al. 2006; Travlos 2006), the hADSCs-Exos were harvested in the conditioned medium from OM of different time-spans (0, 1–14 days, and 15–28 days) in the present study, according to the diverse phases of osteogenesis. The morphology and size distribution of hADSCs-Exos was investigated by TEM and DLS analysis. The exosomes extracted from three stages were not significantly different in size. The extracted hADSCs-Exos all exhibited typical morphology of exosomes and the mean diameter of hADSCs-Exos is 125.8 ± 3.822 nm. Our results revealed the diversity of hADSCs-Exos in morphology, which may relate to their multiple functions in different periods, consistent with previous report (Zabeo et al. 2017). Furthermore, hADSCs-Exos positively expressed exosome-specific protein markers CD9 and CD63 by western blot analysis.

Exosomes can be taken up by recipient cells in the local microenvironment following release. Moreover, hADSCs-Exos implement intercellular communication through target cell internalization (Liu et al. 2019; Tkach and Thery 2016). Fluorescence microscopy analysis showed that the hADSCs-Exo of Exo^0d^, Exo^1−14d^ and Exo^15−28d^ labelled with PKH-67 were taken up by the native hADSCs and mainly distributed in the perinuclear region. Interestingly, we observed that hADSCs-Exos labeled in green were definitely internalized by the hADSCs within 4 h, and this process was high-efficient than that of BMSCs, which taking almost 48 h (Li et al. 2018a). It seems that hADSCs-Exos were more prone to be internalized by hADSCs as their “parent” cells, but not cells from other sources. We speculate that it may be related to the homology of the recipient cells. Numerous direct evidence exists to suggest that the binding of exosomes to recipient cells involves a variety of endocytic pathways, including phagocytosis, macropinocytosis and plasma or endosomal membrane fusion (Mulcahy et al. 2014). The decisive factor for this process may depending on the type of recipient cell and exosomes constituents (Naslund et al. 2014). In another report, they found that only a subpopulation of MSCs internalized exosomes labelled with PKH67, and explained that it may be due to the heterogeneity of MSCs in terms of some certain surface receptors, as well as the different phase of the cell cycle (Wang et al. 2018). Despite all this, the underlying mechanisms of hADSCs-Exo internalized by hADSCs should be further illuminated.

The ability to mineralize is the main functional characteristic of osteoblasts in vitro. hADSCs can be induced to differentiate into osteoblasts that are able to mineralize their extracellular matrix (ECM) and express proteins associated with bone phenotypes (Halvorsen et al. 2001). The osteogenic differentiation capacities of the exosome-treated hADSCs (Exo^0d^, Exo^1−14d^ and Exo^15−28d^) as detected by ARS, the results indicated that hADSCs-Exo^0d^ without osteogenic-induction could not effectively induce hADSCs into osteoblasts in vitro*.* However, both hADSCs-Exo^1−14d^ and Exo^15−28d^ group exhibited remarkable improvement in mineralization of hADSCs. Especially values in the group Exo^1−14d^ were significantly increased in comparison with those in the Exo^0d^, and Exo^15−28d^ group.

During the early stage of osteogenic differentiation, mixed cells at diverse differentiative stages secrete a great quantity of exosomes involving the information exchange of hADSCs, thus establishing a positive-feedback loop during osteogenesis (Cui et al. 2016; Yeo et al. 2013). Actually, in the late stage of osteogenesis, as a result of cellular senescence, contact inhibition and cell differentiation, the capacity of hADSCs secreting exosomes attenuated, meanwhile the function of hADSCs-Exos may also changed (Gudbergsson et al. 2016; Hayes et al. 2005; Li et al. 2018a; Steinman et al. 2003). Some other issues, such as the extraction amount and labor-saving, also need to be taken into consideration for exosomes clinical application in the future. In the present study, the conditioned medium was collected every 3 days, which greatly increased the extraction amount in a limited time. Besides, the time interval of collecting exosomes is 1 to 14 days, which has competitive advantages (e.g. time saving, cost-effective etc.) over prolonged periods. To maximize the efficiency of hADSCs-Exos in osteogenesis, the Exo^1−14d^ was applied to the follow-up research.

Since the proliferative and migratory capacity of transplanted hADSCs are one of the most important processes to promote tissue regeneration (Monaco et al. 2011), we investigated the effects of exosomes derived from osteogenically induced hADSCs on their “parent” cells. After treated with exosomes, the proliferation and cell migration of hADSCs were significantly promoted compared with the negative control group (PM without exosomes). Only Exo^1−14d^ (15 μg/mL) dramatically improved the proliferation of hADSCs at 72 h. Moreover, the migration of hADSCs was enhanced in group Exo^1−14d^ (10, 15 and 20 μg/mL) compared with group PM. In addition, with the increasing concentration of hADSCs-Exos, the cell migratory capacity gradually increased. Moreover, the group Exo^1−14d^ (15 μg/mL) showed the peak of capacity compared with other experimental groups, when the concentration was over 15 μg/mL, the cell migration decreased. We speculate that this may be related to the dysregulation of functional protein activity.

After treated with hADSCs-Exos for 7 and 14 days, ALP staining and activity of hADSCs were significantly enhanced. RUNX2 is a key transcription factor for osteogenesis which acting as early indicators of osteogenesis (Chen et al. 2015; Komori 2003; Komori et al. 1997). Increased expression of RUNX2 as well as ALP activity and mineralization is an essential requisite for bone regeneration(Qi et al. 2016). In our study, RUNX2 and ALP protein expression levels were also analyzed by Western blot. And the results showed the 15 μg/mL hADSCs-Exos after 14 days osteogenically induction significantly promoted the osteogenesis of hADSCs by enhancing ALP activity, extracellular mineralization nodules, and expression of the osteoblastogenesis-related proteins, such as ALP and RUNX2.

The hADSCs-Exos might regulate the osteogenesis of hADSCs by transmitting a variety of signal molecules including mRNAs, miRNAs, non-coding RNAs and proteins to surrounding cells. Takeuchi et al. found that MSC-Exos contain miRNAs that may enhance VEGF secretion from the recipient cells, which contributes to bone regeneration (Takeuchi et al. 2019). Chen et al. demonstrated that exosomes derived from miR-375-overexpressing hADSCs promoted bone regeneration (Chen et al. 2019). However, we did not identify the active component(s) affect osteogenesis in this study, and thus, the regulated mechanism require further investigation.

Considering all of the above experimental results, osteogenically-induced hADSCs-Exos promote hADSCs proliferation, migration and osteogenic differentiation in vitro. hADSCs-Exos at concentration of 15 μg/mL at 1–14 days during osteogenic induction might be optimal. To our best knowledge, this is the first report to apply different stages and concentration of osteogenically-induced hADSCs-Exos to induce the hADSCs osteogenesis in vitro. In conclusion, we suggested that hADSCs-Exo may be an ideal substitute for improving the deficiency of traditional hADSCs therapy in the bone repair. Strategies to load new bone tissue engineering scaffolds with exosomes can be applied for future clinical transformation therapy.

There still existed some limitations about our study. First, the evaluation of bone formation could be performed in vivo. Second, the mechanisms of hADSCs-Exos on promoting osteogenesis and integration of remains further studied.

## Abbreviations

hADSCs: Human adipose-derived stem cells
hADSCs-Exos: Human adipose-derived stem cells-derived exosomes
BMSCs: Bone marrow-derived mesenchymal stem cells
MSCs: Mesenchymal stem cells
TEM: Transmission electron microscopy
PM: Proliferation medium
OM: Osteogenic medium
ARS: Alizarin red staining
DLS: Dynamic light scattering
ALP: Alkaline phosphatase
ECM: Extracellular matrix
EVs: Extracellular vesicles
CCK-8: Cell Counting Kit-8
FBS: Fetal bovine serum
PBS: Phosphate-buffered saline
DAPI: 6-Diamidino-2-phenylindole
RUNX2: Runt-related transcription factor 2
